# Size of Dominant Diatom Species Can Alter Their Evenness

**DOI:** 10.1371/journal.pone.0131454

**Published:** 2015-06-22

**Authors:** Koji Sugie, Koji Suzuki

**Affiliations:** 1 Faculty of Environmental Earth Science, Hokkaido University, Sapporo, Hokkaido, Japan; 2 CREST, Japan Science and Technology Agency, Sapporo, Hokkaido, Japan; American University in Cairo, EGYPT

## Abstract

Traditionally, biodiversity has often been estimated on the basis of abundance partly due to the need for complicated measurements of biomass. Here, we conducted robust measurements of the community composition and of the size structure of diatoms in the North Pacific to evaluate the importance of biomass on the biodiversity. We found that the two most useful evenness indices increased in most cases where small species were numerically dominant when calculations were based on biomass compared with those on abundance. Size-abundance spectra of diatoms revealed that numerically dominant small species rarely dominated in terms of biomass. On the other hand, intermediate to large diatom species generally played a dominant role in terms of biomass in diatom community. The results suggest that the size of the dominant species is a crucial factor in determining the role of diatoms in the ecosystem functioning. Because such size variability can also be observed in other organisms, we need to pay attention to the effect of size structures on biodiversity.

## Introduction

Addressing how the many species in a certain ecosystem coexist is one of the fundamental issues in marine ecology and conservation biology [1]. Ecologists often calculate diversity and evenness indices to assess the relations between environmental conditions and the proportional abundances of each species collected from an environment. Theory is advancing for calculating evenness indices ([2] and references therein) which have been defined as follows:

Evenness=Diversity/Richness. (1)

Richness is the number of species (*S*) or the number of traits. There is increasing evidence that the productivity and stability (or vulnerability) of a marine ecosystem can be influenced by biodiversity [3, 4]. Evenness is often sensitive to environmental changes, such as anthropogenic perturbations and climate changes, compared with richness, suggesting that evenness may be one of the factors important to ecosystem functioning [5, 6]. However, to date, there has been very limited information available concerning the biodiversity of marine phytoplankton, specifically diatoms, in the North Pacific [7]. Furthermore, methods for calculating diversity and evenness indices in plankton communities vary among reports; one major differences is that the calculation of some indices are based on abundance, i.e., cell density [8] and some are based on biomass [9]. We hypothesize that such inconsistencies may lead to different conclusions on the biodiversity of plankton communities due to the large variability in the cell sizes of phytoplankton.

Marine phytoplankton are responsible for nearly half of global primary production, thereby forming the base of marine ecosystems [10, 11]. The biomass, diversity, community composition and size structure of phytoplankton are important factors influencing the productivity and biogeochemical cycling of bio-elements in the ocean [4, 12]. The cell size of phytoplankton is a prime trait that affects almost all aspects of marine ecology, such as the metabolic rate, affinity for nutrient uptake, sinking behavior and biotic interactions [13, 14]. Generally, the size spectra of a plankton community, including bacteria, phytoplankton and heterotrophic protists can be described by a power function that represents abundance as decreasing exponentially with increasing cell size [15, 16]. This trend clearly indicates that plankton communities should represent low evenness when calculated on the basis of abundance. However, one large cell contains more carbon and nutrients and occupies a larger biovolume per unit seawater mass than one small cell. It is well documented that numerically less dominant species with large cell sizes often dominate in terms of biomass, particularly in high-production environments [17, 18]. A recent study suggested that the mean cell size in a phytoplankton community changed in response to the change in oceanographic conditions [16, 19]. Therefore, the calculation of diversity and evenness indices based on cell density needs further attention because of the lack of information on cell size.

According to Hasle and Syvertsen [20], the cell volume of plankton specifically diatoms varies by ~9 orders of magnitude, ranging from ~2 (e.g., *Minutocellus* and *Arcocellulus* spp.) to ~6 × 10^9^ μm^3^ (e.g., *Ethmodiscus* spp.). Diatoms are one of the most prominent phytoplankton groups in the modern seas, and they can contribute to ~40% of primary production in the global ocean [10, 11]. In general, a given amount of nutrients can support high abundance of smaller cells compared to larger ones, following the allometric log-log linear relationship between cell size and nutrient cell quota [21]. Relationships between cell size and abundance of natural plankton community could be characterized by the ecophysiology of each plankton species [18]. Recent studies suggested that the cell size-dependent change in nutrient and light use strategy with nutrient and light availability may characterize the spatiotemporal patterns of size-abundance spectrum [14, 16, 18, 19]. The specific growth rates of diatoms decrease as their cell size increases [14, 22], suggesting that the abundance of larger cells is lower than that of smaller cells. In addition, the affinity for nutrient uptake is generally higher in smaller cells than that in larger relatives due to the difference in surface area to cell volume ratio and in the size of diffusive boundary layer [22, 23, 24]. However, large or heavy silicified diatoms have the potential to resist grazing by micro- and meso-zooplankton [25, 26, 27]. These ecological tradeoffs make diatoms with smaller cells resource competition specialists and those with larger cells predator-defense specialists [26]. Modeling studies have suggested that the size structure and diversity of a phytoplankton community are affected by such ecological tradeoffs [28, 29]. As a result, the lack of information on cell size can overlook the importance of such tradeoffs on the role of diatoms in the ecosystem functioning.

Here, we show the differences between biomass- and abundance-based estimates of evenness of diatoms in the North Pacific to help us determine the importance of using biomass data rather than abundance data for estimating evenness. Second, we analyzed the size structure of diatoms to explore the mechanisms that affect evenness indices and to address probable relations between cell size and the dominance of diatoms in the ocean.

## Materials and Methods

### Ethic statements

No specific permissions were required for sampling in the high seas. None of sampling stations was located within a nature reserve (S1 Table) and samples used in this study were not involved endangered or protected species. The samplings carried out in the exclusive economic zones (EEZ) in the USA and the Federated States of Micronesia, we followed the rules of each government which provided for each research cruises.

### Sampling strategy

**Fig 1:**
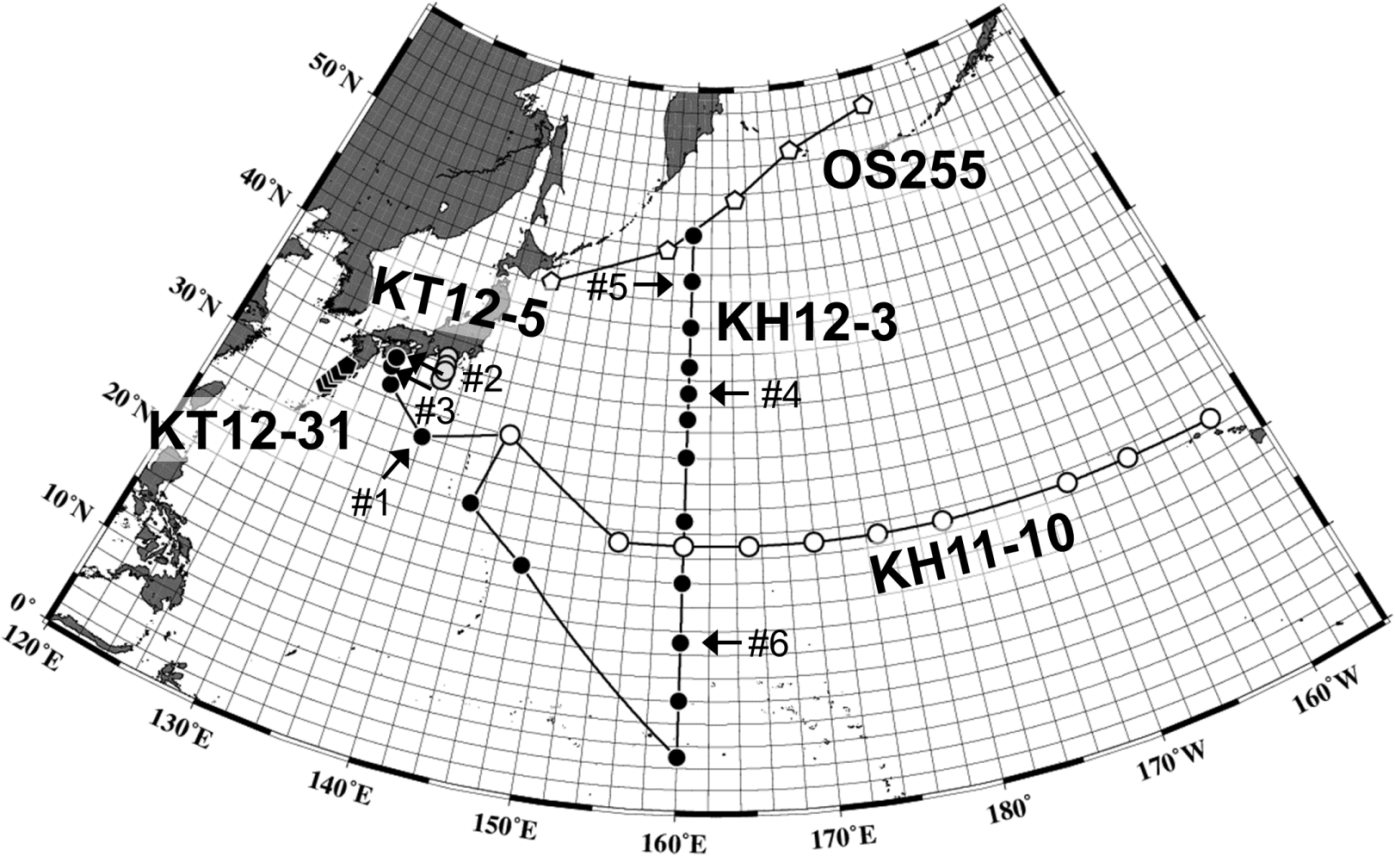
Sampling stations (circles) in the North Pacific with the cruise names. White circle: R/V *Hakuho-maru*, KH11-10 cruise; Black circle: R/V *Hakuho-maru*, KH12-3 cruise; Light gray circle: R/V *Tansei-maru*, KT12-5 cruise; Dark gray circle: R/V *Tansei-maru*, KT12-31 cruise. Arrows with a #number represent the sample IDs shown in Fig 2.

Samples were collected from the North Pacific region (Fig 1). Twenty samples were collected during the R/V *Hakuho-maru* KH11-10 cruise on December 2011; 34 during the KH12-3 cruise on July–August 2012; 12 during the R/V *Tansei-maru* KT12-5 cruise on April 2012; 10 during the KT12-31 cruise November 2012; 5 during the T/S *Oshoro-maru* OS255 cruise on June 2013 (S1 Table). Seawater was collected from the surface (0, 5 or 10 m) and from subsurface chlorophyll maximum layers. For inverted light microscope analysis, acid Lugol’s iodine solution was added to 500 mL of seawater at a final concentration of 4% and 500 mL of seawater was analyzed according to the method of Hasle [30]. For scanning electron microscope analysis, samples were fixed with 10% paraformaldehyde (pH 7.4) at a final concentration of 0.1%. These samples were stored in a refrigerator until on land analysis. Diatom species were identified according to Round et al. [31], and Hasle and Syvertsen [20] and their references. The cell volume of diatom species was measured using the geometric shapes described by Hillebrand et al. [32], and the cell volume was converted to carbon biomass using the allometry reported by Menden-Deuer and Lessard [21]. Because diatoms have large, carbon-poor vacuoles [33], biomass rather than cell volume was used to explore the importance of diatom to the marine ecosystem, such as in their trophic interactions.

### Calculation of evenness indices

**Fig 2:**
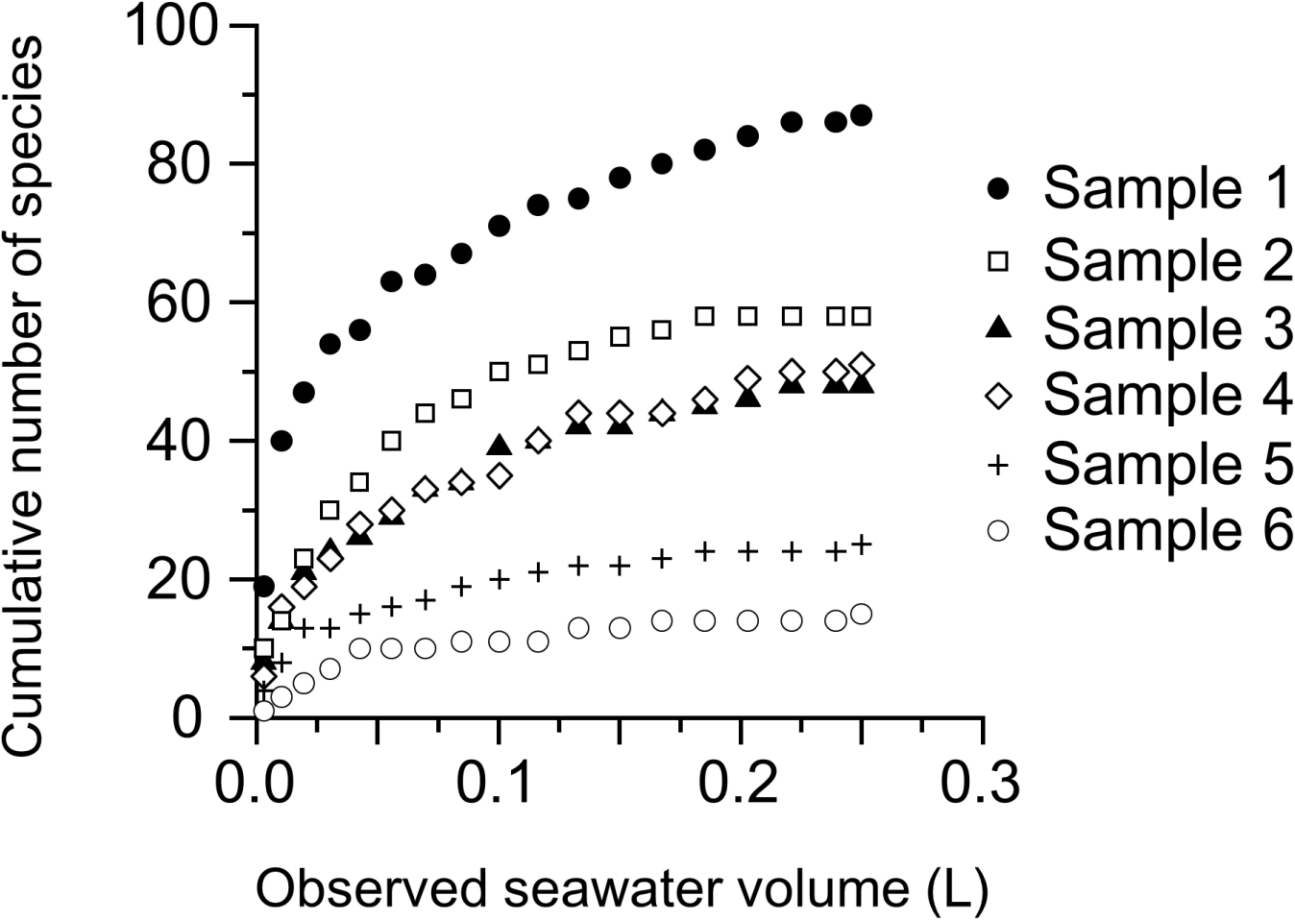
Species accumulation curves of 6 samples obtained during the KH12-3 cruise (see Fig 1). Sample information is provided as follows: sample 1: 5 m, 28°00’ N, 138°00’ E; sample 2: 75 m, 32°19’ N, 133°33’ E; sample 3: 43 m, 33°05’ N, 133°40’ E; sample 4: 75 m, 35°00’ N, 160°00’ E; sample 5: 5 m, 43°31’ N, 160°00’ E; sample 2: 75 m, 15°00’ N, 160°00’ E.

The two most widely-used diversity indices applied in Eq 1 are the Shannon-Weiner (*H*′) and Simpson (*λ*) indices [2, 34]. These are calculated as follows:

$$H\prime =-\sum_{i=1}^{S}p_{i}\text{Ln}(p_{i})$$ (2)

$$\lambda =\sum_{i=1}^{S}p_{i}^{\;2},$$ (3)

where *p*_*i*_ represents the proportional abundance of the *i*th species (or traits of interest) and *S* represents the number of species or species richness. In this study, we used the number of species for *S* values which is referred to as species density in a strict sense [35]. Comparing the above two diversity indices, *λ* is weighted more for the most abundant species compared to *H*′ [2]. In this study, we used the following evenness indices, which are determined using the above two diversity indices as follows:

$$J\prime =\frac{H\prime }{H\prime {}_{\text{max}}}$$ (4)

$${}^{2}E=\frac{1}{\lambda \times S},$$ (5)

where *H*′_max_ is ln(*S*) [36]. *J*′ is defined entropy ratio, which is useful and widely applied in many ecosystems [2]. ^*2*^ *E* is one of the best indices for fulfilling the essential requirements of the definition of evenness [34]. Because most diversity indices are derived from *p*_*i*_ [2], we believe that the results obtained in this study have generality. Hereafter, *J′* and ^*2*^ *E*, which are based on abundance, are referred to as *J′*_cell_ and ^*2*^ *E*_cell_. When biomass data were applied to the calculation of evenness indices, *p*_*i*_ values in Eqs 2 and 3 were modified as proportional biomass, i.e., the biomass of the *i*th species per total biomass of diatoms in a sample. Hereafter, *J′* and ^*2*^ *E*, which are based on biomass, are referred to as *J′*_bio_ and ^*2*^ *E*_bio_. Note that it is widely recognized that *S* is strongly affected by sampling effort e.g., [37, 38]. To validate the *S* value used in the present study, we measured the species accumulation curves of data from the light microscope, mainly from the samples collected on the KH12-3 cruise. Some typical examples shown here are nearly saturated with diatom species within a 250 mL of seawater samples (Fig 2), suggesting that the *S* values used for Eqs 4 and 5 in this study were representative of the diatom community richness of the examined seawater. However, recent studies have implied that there may still not be enough to detect all rare species because of the nature of the heterogeneous distribution of organisms in the ocean [38, 39].

To explore the mechanisms that affect evenness and to show the relations between cell size and the dominance of diatoms, the size spectrum of biomass and abundance with corresponding cell volume were analyzed according to the method of Marañon [16]. To normalize the difference of total biomass and abundance among samples, the relative biomass/abundance of each species in each sample were calculated by dividing the biomass/abundance of a single species by total biomass/abundance of diatoms in a sample. After pooling all data of relative biomass and abundance, size classes were constituted on an octave scale (log_2_) of cell volume. The total of relative biomass and abundance of each size class were pooled and analyzed using boxplots to obtain the trends of size spectrum of relative biomass and abundance with a statistical variability. To further explore the size variations, cell volume of each diatom species was converted to equivalent spherical diameter (ESD). In addition, the pooled data of relative biomass/abundance were grouped into 1–0.1, 0.1–0.01, 0.01–0.001 and <0.001 categories. The histograms of each group were developed using 2 μm ESD bin to examine the dominancy-dependent change in size composition. Diatoms with >100 μm in ESD were grouped together because of their low frequency of occurrence in a 500 mL seawater sample. Histograms were statistically analyzed by using the software Origin (version 8.0, OriginLab Corp., Northampton, MA, USA) to fit a Gaussian function. Size diversity was calculated from the histogram data treated with one size bin as one species and occurrence as abundance using the Shannon-Weiner formula (Eq 2), and representing the diversity as *H′*_size_.

## Results

**Fig 3:**
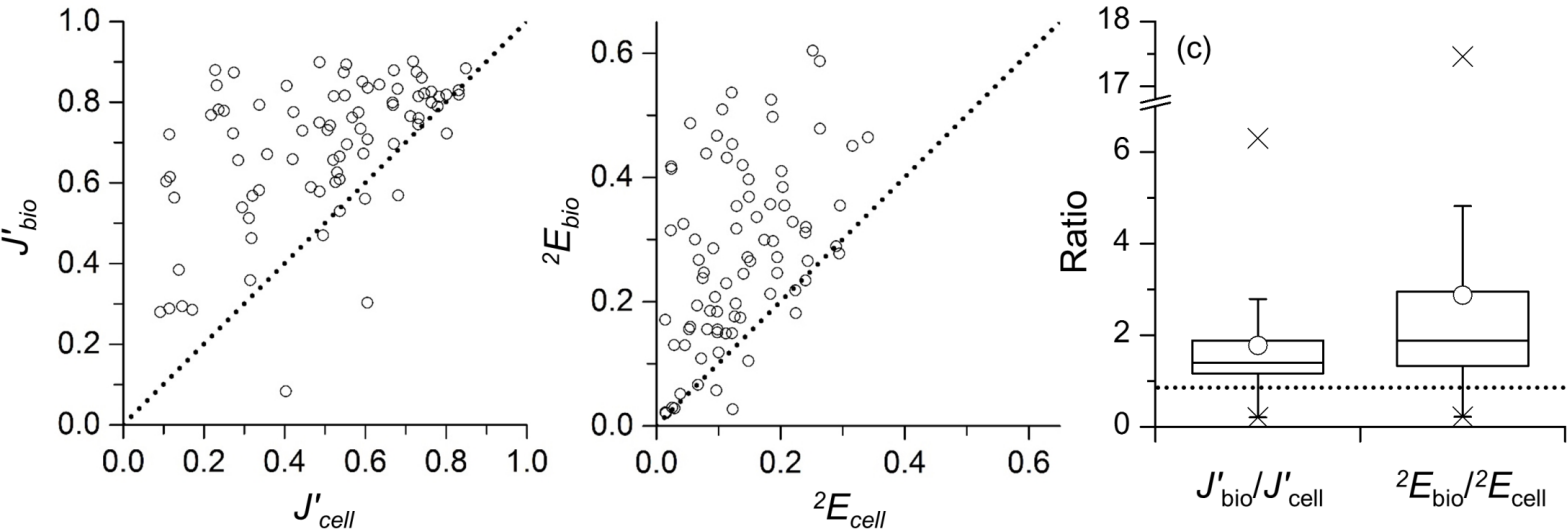
Relationships between (a) *J′* calculated based on abundance (*J′*_cell_) and biomass (*J′*_bio_), and (b) *^2^E* calculated based on abundance (*^2^E*_cell_) and biomass (*^2^E*_bio_). The dotted lines in (a) and (b) represent the 1:1 line. (c) Boxplot of *J′*_bio_/*J′*_cell_ and ^*2*^ *E*_bio_/^*2*^ *E*_cell_. The upper and lower lines of the box represent the 75% and 25% values. The middle line in the box and circle represents the median and average of the ratios. Crosses represent maximum and minimum values. The ratio = 1 (dotted line), has the same meaning as the 1:1 line shown in Fig 3a and 3b.

It is obvious that a large number of data were plotted above the 1:1 line for both evenness indices (Fig 3), indicating that the evenness of diatoms increased as calculated with biomass compared with abundance. The large increase of evenness indices based on biomass was associated with the samples where small diatoms such as *Arcocellulus*/*Minutocellus* complex, *Minidiscus trioculatus*, and *Nitzschia bicapitata* complex (ESD: ca. 2, 3.5, and 5 μm, respectively) dominated in terms of abundance. However, it should also be noted that 8 out of 81 *J′*, and 10 out of 81 ^*2*^ *E* were located beneath the 1:1 line (Fig 3A–3C). In the latter cases, extremely large diatoms such as *Coscinodiscus* spp. (ESD: > 100 μm), *Neocalyptrella robusta* (ESD: ~250 μm), *Rhizosolenia clevei* (ESD: ~280 μm) and *Rhizosolenia imbricata* (ESD: ~230 μm) dominated in terms of biomass.

**Fig 4:**
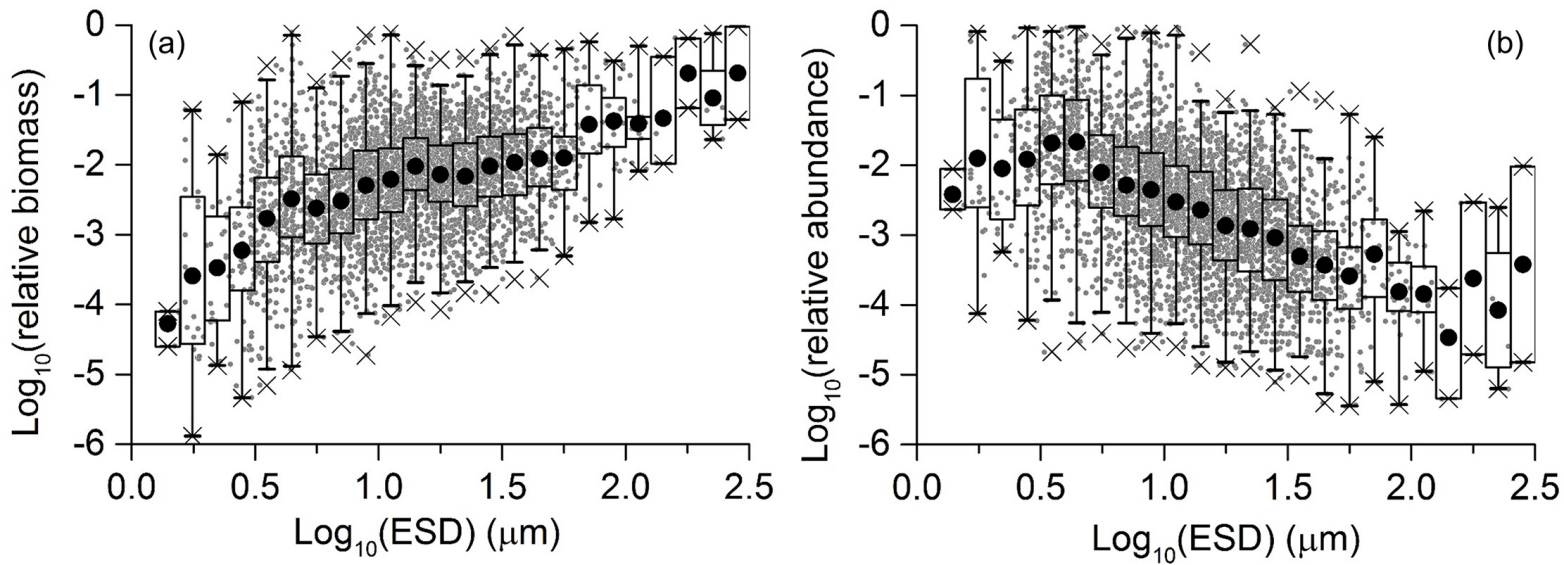
(a) Biomass-ESD and (b) abundance-ESD spectra of diatoms in the North Pacific. Scattered diagrams (gray dots) represent the relationships between relative (a) biomass or (b) abundance and ESD of pooled samples. Boxplot represents the (a) biomass and (b) abundance of the each size class, established in an octave (log_2_) scale. Black circle, box, bars, and crosses represent the mean values of each group, ranges for 25–75% of data, ranges for 1–99% of data, and outliers, respectively.

We found that the relative biomass increased rapidly with increasing cell size from ~1 to ~5 μm in ESD and steadily increased with increasing cell size > 5 μm in ESD (Fig 4A). The relative biomass of extremely large cells (ESD: > 100 μm) tended to dominate in a diatom community once they occurred even with low abundance (< ~50 cells L^−1^). However, note that the frequency of occurrence of such large species was relatively low and the data error could potentially be large. When extremely large cells were absent in a sample, intermediate-sized species tended to dominate in a diatom community in terms of biomass (Fig 4A). In contrast to the relative biomass-cell volume spectrum, relative abundance-ESD spectrum showed a unimodal distribution, with a peak around 3–5 μm in ESD (Fig 4B). The relative abundance monotonously decreased with increasing cell size > 5 μm in ESD.

**Fig 5:**
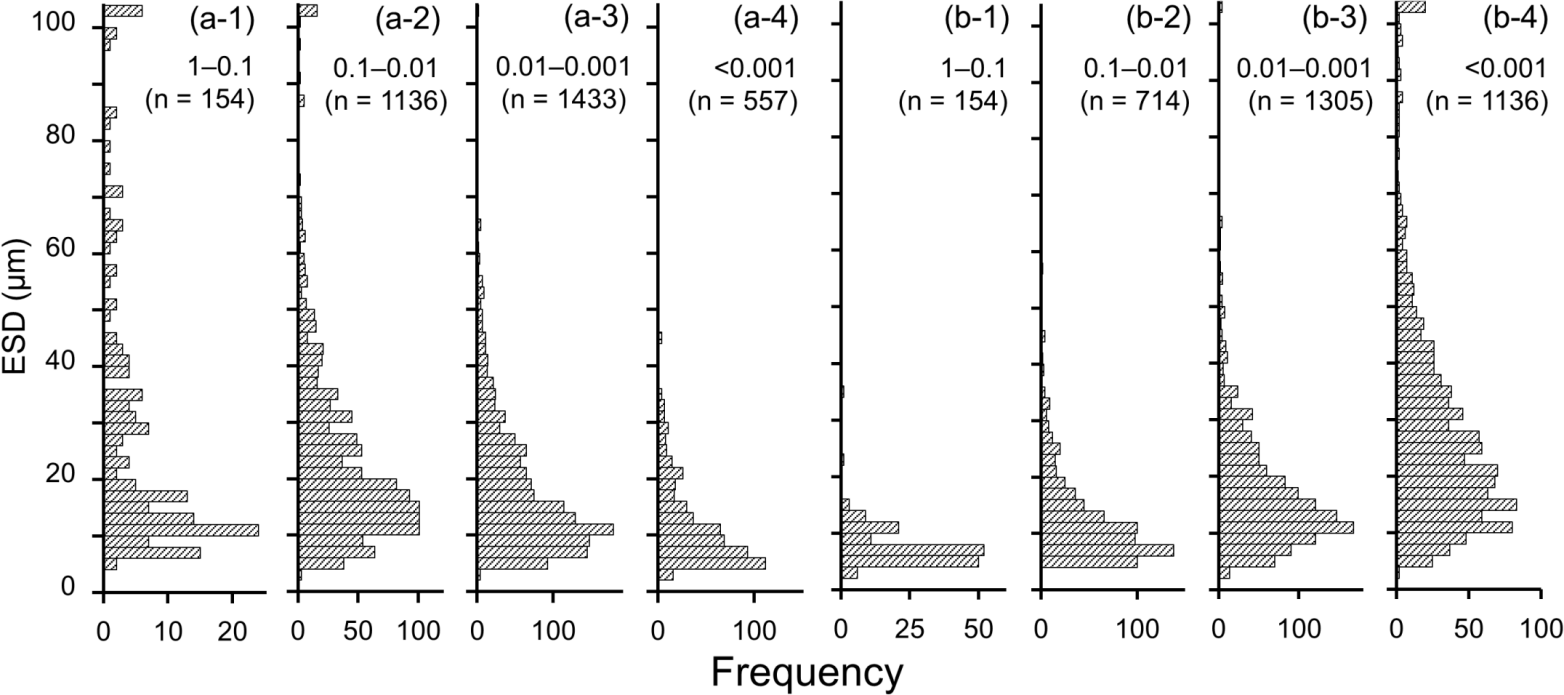
Histograms represent the ESD distribution based on the relative (a) biomass or (b) abundance shown in Fig 4. Pooled data were assembled into (a-1) and (b-1) 1–0.1, (a-2) and (b-2) 0.1–0.01, (a-3) and (b-3) 0.01–0.001, and (a-4) and (b-4) <0.001 of relative biomass or abundance.

As estimated from the histogram analysis, small-sized species (< 10 μm in ESD) contributed high frequency in the dominant fractions (> 0.01) of relative abundance (Fig 5B-1 and 5B-2), but they observed in the > 0.01 fractions of the relative biomass with low frequency (Fig 5A-1 and 5A-2). Intermediate-sized species (10–60 μm in ESD) were frequently found in the 1–0.001 fractions of relative biomass (Fig 5A-1–5A-3), whereas their occurrence were generally restricted in the < 0.01 fractions of relative abundance (Fig 5B-3 and 5B-4). Extremely large-sized cells were not found in the dominant fraction of relative abundance, but it appeared in the 1–0.1 fraction of relative biomass (Fig 5A-1 and 5B-4). More than 78% of the frequency of occurrence of relative biomass accounted for the fractions of intermediate dominancy (0.1–0.001), whereas most of the frequency of relative abundance accounted for the rare fractions (< 0.01). The histogram of the relative biomass-based analysis revealed that the size range (*w*) and mean cell size (*m*) increased from the group of 1–0.1 to 0.1–0.01, and these were slightly lower in minor groups of relative biomass (Fig 5A, Table 1). In the case of relative abundance-based analysis, the largest *w* and *m* values were observed in the rarest (< 0.001) group (Fig 5B, Table 1). The values of size diversity index (*H′*_size_) were slightly higher in the groups of dominant relative biomass, whereas the *H′*_size_ based on relative abundance estimates showed the highest in the rarest group (Table 1).

**Table 1:** Statistical results of the range of size (width parameter of the Gauss distribution: w, μm) and the mean cell size in the diatom community (m: μm) fitted by the Gaussian function shown in Fig 5.

Contribution	*w*	*m*	Statistic values	*H′*_size_
Biomass-base				
1–0.1	7.7 ± 1.1	10.7 ± 0.5	*p* < 0.001, R^2^ = 0.65	3.17
0.1–0.01	15.0 ± 1.2	14.5 ± 0.5	*p* < 0.001, R^2^ = 0.86	3.19
0.01–0.001	11.6 ± 1.0	10.7 ± 0.5	*p* < 0.001, R^2^ = 0.84	2.92
<0.001	7.1 ± 0.6	6.7 ± 0.3	*p* < 0.001, R^2^ = 0.83	2.52
Abundance-base				
1–0.1	3.1 ± 0.2	5.1 ± 0.1	*p* < 0.001, R^2^ = 0.90	1.63
0.1–0.01	7.7 ± 0.6	7.9 ± 0.3	*p* < 0.001, R^2^ = 0.87	2.44
0.01–0.001	12.2 ± 0.7	12.0 ± 0.3	*p* < 0.001, R^2^ = 0.92	2.88
<0.001	23.9 ± 1.8	19.2 ± 0.8	*p* < 0.001, R^2^ = 0.87	3.41

Data represent the mean ± 1SE of regression. *H′*_size_ represents the size diversity shown in Fig 5.

## Discussion

We found large differences in the evenness indices generated from abundance- and biomass-based estimates, and these differences were due to differences in the sizes of the dominant diatom species. In Eqs 2 and 3, when diversity and evenness indices are calculated on the basis of biomass, the contribution of *p*_*i*_ in the diversity indices of numerically dominant small-cell species usually decreases, whereas those of large-cell species increases, resulting in a change of evenness. We showed that intermediate to large size (ESD: > 10 μm) species frequently dominated in terms of biomass whereas numerically-dominating small (ESD: <5 μm) species contribute small in terms of biomass. Such a pattern of dominance contributed to an increase in the *p*_*i*_ of the intermediate to large size species. Further, the size histogram of the relative biomass exhibited a broad unimodal distribution of ESD, indicating that many species of different cell sizes could dominate in the diatom community in the North Pacific. Such size variability in the dominant species can lead to an increase in *J′*_bio_ and ^*2*^ *E*_bio_ than *J′*_cell_ and ^*2*^ *E*_cell_, respectively, unless extremely large diatoms dominate (see below). From an ecological point of view, dominance of variable size in phytoplankton may support the growth of many herbivores in a greater range of size. Further, because dominance in terms of biomass, in other words cell volume, represents the degree of the occupancy of space niche, evenness and diversity indices based on biomass have a clear advantage considering the importance of biodiversity in an ecosystem. It has been well documented that the slopes of the size-scaling of plankton abundance vary spatiotemporally, reflecting the size of the dominant species in the ocean [16, 18, 19]. In both ocean and lake systems, diatom cell size can be affected by macro- and micro-nutrient availability as well as *p*CO_2_ or pH [28, 40, 41, 42]. Therefore, we suggest that the evenness and diversity indices of diatoms should be calculated based on biomass to determine the relations between biodiversity and environmental conditions.

We found that evenness indices based on biomass were relatively low in ca. 10% of the samples where extremely large (> 100 μm in ESD) diatom species dominated compared with those based on abundance. The dominance of large species, such as *Rhizosolenia* spp. have been found in oligotrophic subtropical waters, which probably resulted from its association with diazotrophic cyanobacteria. With such a symbiotic relationship, a diatom species may be able to survive and dominate under nutrient-depleted conditions, even with a large cell size and a slow specific growth rate [43]. Other underlying mechanisms for the dominance of large diatom species may be hard to specify. In general, large-cell phytoplankton do not have an advantage compared to the small-cell species with respect to nutrient uptake and sinking behavior in the open ocean, where macro- and/or micro-nutrient depleted [14, 22, 26, 44]. Some large diatoms may be able to store nutrients in their vacuoles [33], escape from small grazers [24, 26], fix carbon faster than small species [16, 18], and migrate between the sunlit surface and the nutricline [45]. However, the survival strategies of large diatoms and their success in the open ocean is still one of the fascinating unresolved questions about the ecology of diatoms.

In contrast to the dominance of intermediate and large diatom species, small species rarely dominated in the community in terms of biomass despite their potentially faster growth rate, high efficiency for light use and nutrient acquisition, and slower sinking rate [14, 18, 22, 41]. In addition, we found very small diatoms (< ~3 μm in ESD) are not dominant even in terms of abundance. Some tychopelagic species with small cell size such as *Cyclotella atomus* and *Psammodictyon panduriforme* apparently contributed little to biomass, probably because their habitat would primarily be in a benthic environment [31, 46]. However, other small holoplanktonic species, such as *Lennoxia faveolata* and *Minidiscus trioculatus*, often made small contributions in terms of biomass. Possible mechanisms underlying the rarity of small holoplankton are as follows: (i) faster growth rate with a higher mortality from a high grazing pressure compared to the larger species [25, 26]; (ii) strain-, species- or genus-specific differences in growth rate depending on environmental variability, such as light regime and trace metals [47, 48]; and (iii) the unexplored unimodality of diatom growth rate, i.e., the growth rate of an extremely small diatom is slower than that of an intermediate one, which was recently discovered in marine phytoplankton in a study of five phyla [14, 18].

We found that diatoms of intermediate-size (>10 μm) frequently outcompeted small size classes in terms of biomass except where extremely large-cell species dominated. Recent study hypothesized that the balance among cell size-dependent changes in maximum nutrient uptake rate, nutrient requirement, light use efficiency, and the cost of intracellular metabolism could become a key to determine the growth rate and size structure of phytoplankton community [18, 19]. However, this fascinating hypothesis is based on the values obtained from laboratory cultures under nutrient-replete condition, whereas most of examined area in this study were located in macro- and/or micro-nutrient depleted environment [44]. One of the other possible reasons for the dominance of intermediate-sized diatoms is chain- and mucilaginous colony-formation. It has been well documented that chain-forming diatom species often produce massive blooms in coastal waters e.g., [49, 50]. Thingstad et al. [26] hypothesized that diatoms, in general, simultaneously achieved high affinity for nutrient uptake and predator defense, with their Si cell walls serving as a survival strategy, i.e., large yet small strategy, which may lead them to success in modern seas. The formation of long-chains and/or large-colonies decreases grazing pressure and may further enhance the bloom of diatoms [25, 26]. Fawcett and Ward [51] demonstrated that bloom-forming diatoms such as the chain-forming *Chaetoceros* species responded quickly to nutrient pulses, and then dominated in terms of biomass. However, the chains would have had faster sinking rates and a lower diffusive nutrient supply than the smaller, solitary cells. Therefore, the strategy of chain-formation may be restricted by high turbulence and nutrient conditions, such as those found in coastal regions [23]. Unfortunately, such chains are frequently broken during treatment for microscopy, mainly during fixation. It is essential to know the apparent size of diatom chains to better understand the size-dominance relation and thus the evenness and diversity of diatoms in the ocean. However, the dominance of intermediate-size species in this study was found in the macro- or micro-nutrient depleted surface layers in the subarctic and subtropical North Pacific. These evidences suggest that not only chain or colony formation but also other unexplored mechanisms would help to dominate intermediate-size diatoms in the ocean.

Overall, we demonstrated that the evenness indices for diatoms as estimated with biomass clearly differed from those estimated with abundance. Our results provide insights into many organisms of interest that have a large variability in the size of each species or in traits specific biomass, such as mammals (mouse to elephant or blue whale), Insecta (fairyfly to beetle), and woody plants (sprout to old tree). Further, functional diversity indices are computed based on the traits per individual or species [1, 52]. Because the weighting of traits is one of the key problems for estimating functional diversities e.g., [52], we propose that ecological traits should be weighted using biomass when the species or traits of interest have a large variability in the size of cells or individuals. Many previous studies explored the relations between diversity indices and ecologically relevant factors, such as productivity and invasibility, but the trends are inconsistent among reports [1, 6]. We need to reinvestigate these relations with deliberate attention being paid to the size of the organisms of interest.

## Supporting Information

S1 Table Cruise name, sampling date, station name, latitude, longitude, sampling depth, temperature. (DOCX)
