# Supplementary material for: Size of Dominant Diatom Species Can Alter Their Evenness
Source: PLoS One. 2015 Jun 22;10(6):e0131454. doi: 10.1371/journal.pone.0131454 (PMC4476557; doi:10.1371/journal.pone.0131454)
Supplement: S1 Table — (DOCX) [file pone.0131454.s001.docx]

Table S1. Cruise name, sampling date, station name, latitude, longitude, sampling depth, temperature.

Cruise Date Stn. Lat. Lon. Depth Temp.

(d/m/y) (N) (m) (ºC)

KH11-10 3/12/2011 0 30º00' 145º00' E 5 23.8

90 19.7

5/12/2011 1 23º00' 155º00' E 5 26.9

130 20.0

7/12/2011 2 23º00' 160º00' E 5 26.7

120 20.3

10/12/2011 3 23º00' 165º00' E 5 27.4

120 24.1

12/12/2011 4 23º00' 170º00' E 5 26.4

110 19.0

13/12/2011 4b 23º00' 174º55' E 5 26.3

110 22.6

13/12/2011 5 23º00' 180º00' 5 26.4

110 22.7

16/12/2011 6 23º00' 170º00' W 5 25.5

110 23.2

17/12/2011 7 23º00' 165º00' W 5 25.1

120 23.5

19/12/2011 8 22º45' 158º00' W 5 24.3

100 24.2

KT12-5 9/4/2012 C1 34º30' 138º00' E 5 15.9

30 15.3

10/4/2012 C5 33º31' 138º04' E 5 21.4

80 19.0

11/4/2012 C7 33º51' 138º05' E 5 18.6

35 18.5

13/4/2012 C8 32º10' 138º00' E 5 21.7

90 20.0

13/4/2012 C10 32º42' 138º00' E 5 21.1

35 21.0

14/4/2012 C12 33º23' 138º00' E 5 22.0

40 21.8

KH12-3 10/7/2012 1 46º56' 159º59' E 5 8.7

43 2.7

11/7/2012 2 43º30' 160º00' E 5 13.9

37 10.9

13/7/2012 3 40º00' 160º00' E 5 17.3

40 12.7

14/7/2012 4 37º00' 160º00' E 0 22.0

15/7/2012 5 35º00' 160º00' E 5 24.0

75 19.0

16/7/2012 6 33º00' 160º00' E 0 24.7

17/7/2012 7 30º01' 159º59' E 5 27.3

82 18.2

18/7/2012 8 25º00' 160º00' E 5 29.1

138 18.3

20/7/2012 9 20º00' 159º59' E 5 29.2

125 23.1

22/7/2012 10 15º00' 160º00' E 5 29.2

140 25.8

23/7/2012 11 10º00' 160º00' E 5 29.2

130 25.0

24/7/2012 ExS 5º00' 160º00' E 5 28.3

2/8/2012 12 20º00' 148º00' E 10 28.9

108 21.4

5/8/2012 14 24º00' 142º13' E 5 28.7

100 22.0

6/8/2012 13 30º00' 144º50' E 0 27.3

8/8/2012 15 28º00' 138º00' E 5 27.8

91 20.2

9/8/2012 16 31º00' 134º04' E 5 28.0

105 19.7

10/8/2012 17 32º19' 133º33' E 5 29.0

75 23.4

11/8/2012 18 33º05' 133º40' E 5 28.6

43 20.3

KT12-31 19/11/2012 1 29º00' 129º00' E 5 24.2

60 24.2

20/11/2012 2 29º50' 129º15' E 5 24.1

60 24.1

20/11/2012 3 30º00' 129º30' E 5 24.3

60 24.3

21/11/2012 4 30º20' 129º40' E 5 23.3

40 23.3

22/11/2012 6 31º00' 130º00' E 5 21.7

35 21.4

OS255 14/6/2013 1 42º09' 145º32' E 5 13.4

16/6/2013 2 45º42' 157º11' E 5 9.6

18/6/2013 3 49º31' 164º40' E 5 7.7

19/6/2013 4 52º55' 171º48' E 5 8.0

20/6/2013 5 55º12' 177º41' W 5 8.1
